# Investigating determinants of catastrophic health spending among poorly insured elderly households in urban Nigeria

**DOI:** 10.1186/s12939-015-0188-5

**Published:** 2015-09-15

**Authors:** Olumide Adisa

**Affiliations:** School of Sociology, and Social Policy, University of Nottingham, University Park Campus, Nottingham, NG7 2RD UK

**Keywords:** Catastrophic health expenditures, Informal health care financing, Poverty, Elderly households, Urban Nigeria

## Abstract

**Background:**

In the absence of functional social security mechanisms for elderly people in Nigeria, elderly households are solely responsible for geriatric healthcare costs, which can lead to catastrophic health expenditures (CHE) – particularly among the poor. This study investigates the key determinants of CHE among poorly insured elderly households in Nigeria. We also offer some policy options for reducing the risk of CHE.

**Methods:**

Data on out-of pocket payments and self-reported health status were sourced from the Nigerian General Household Panel Survey (NGHPS) in Nigeria, conducted by the National Bureau of Statistics in 2010, with technical support from the World Bank. CHE was defined at the 10 % of total consumption expenditure threshold. The determinants of CHE and their marginal effects were investigated using probit regressions. An elderly household is defined as a household with at least one elderly member ≥ 50 years old.

**Results:**

The proportion of elderly households with CHE is 9.6 %. Poorer and smaller elderly households were most at risk of CHE. Female-headed households were less likely to incur CHE compared to male-headed households (*p* < 0.01). Conversely, households with informal health financing arrangements were less likely to incur CHE (*p* < 0.001). Education and utilising a health promoting tool, such as treated bednets increased the probability of incurring CHE in Urban Nigeria.

**Conclusion:**

Findings from this paper should prompt policy action to financially support poor elderly households at risk of CHE in Urban Nigeria. The Nigerian government should enhance the national health insurance scheme to provide better coverage for elderly people, thereby protecting elderly households from incurring CHE.

## Introduction

Catastrophic health expenditures (CHE) have negative welfare implications on households [1–4]. In a survey of 89 countries, Xu et al. further reported higher levels of CHE in low and middle income countries (LMICs) in comparison to developed high income countries. Globally, over 150 million people incur CHE and approximately 100 million people become impoverished as a result [4]. Researchers at the World Health Organisation (WHO) propose that health spending becomes catastrophic when out-of-pocket health spending exceeds 15-20 % of total health expenditure level [4, 5]. Other studies have proposed CHE definitions based on health spending greater than or equal to 10 % of household expenditure [1, 6] or 40 % of non-food household expenditure [4, 5, 7]. Although these baseline levels differ in the literature, most studies agree that the incidence of CHE is higher in LMICs than in developed countries.

Like many LMICs, OOP (out-of-pocket payments) are the most prevalent method of financing health care costs in African countries. In Nigeria, over 70 % of health spending is private, and 96 % of private health expenditure is made up of out-of pocket payments [8]. In many developing countries, the principal use of OOP to finance health care has spurred a growing body of research regarding the existence and determinants of CHE among households [2, 4, 5, 9–12]. In Nigeria, the existence of CHE was confirmed by Onoka et al. in their 2011 study. The authors reported that about 40 % of households in South Eastern Nigeria incurred health costs greater than 10 % of their consumption expenditure.

Unfortunately, very few studies focus specifically on CHE among elderly households in LMICs, with the exception of Wang et al. in China [11]. However, some studies in other developing countries have found that having an elderly person in the household pre-disposes households to CHE. For instance, Xu et al. found that elderly households, households with disabled or chronically ill members were more at risk of catastrophic health spending than other households [4]. Wagstaff and Doorslaer, and Somkotra and Lagrada also found similar results in Vietnam and Thailand respectively [1, 12].

Furthermore, rapid population ageing has intensified concerns about the extent to which geriatric health spending can become catastrophic, particularly in African countries with minimal social welfare policies for elderly people [13, 14], and Nigeria is no exception. Nigeria has a small but growing elderly population representing 4 % of the 174 million population—approximately 7 million people [15].^1^ In 2014, Nigeria’s social security health expenditure for the elderly was reported as negligible by the WHO [16]. In recent years, many elderly people remain excluded from the national health insurance scheme (NHIS) in Nigeria [17], have little or no pensions and income generating opportunities [18, 19]. Therefore, identifying the associated factors of CHE among elderly households in Nigeria is necessary—more so, for urban residents.

While it is reasonable to assume that the risk to CHE differs somewhat amongst urban households, the vast majority of urban elderly households in Nigeria are ageing in the midst of high levels of poverty. Using the National Living Statndards Survey 2003/2004 from Nigeria, Appleton et al. put poverty levels in urban areas at around 50 % [20]. In our study, crude estimates put 70 % of urban elderly households in poverty at the World Bank’s poverty line of $1.25 per day. Another characteristic of the urban Nigerian environment is that when residents are ill, they face high medical costs from private-to-profit health organisations [21, 22]. These private health organisations currently provide 80 % of health services in Nigeria [17], and commentaries from health observers suggest that this trend is likely to continue. For instance, Ogunbekun et al. testify that the heavy dependence on private health facilities among low-income urban residents in Nigeria is likely to remain so long as the quality of government health services remains appalling [22].

Furthermore, later-life health studies on the Nigerian elderly suggest that elderly groups experience a decline in physical and mental capabilities unique to old age, which increases dependence for care [17–19, 21–23]. In one community survey of elderly Nigerians aged 60 years and above, Bella et al. found that the most common health problems of elderly people were ‘musculoskeletal, dental, ocular and cardiovascular’ diseases [23], which lead to higher demand for care. A cross-country study of elderly Nigerians in the South West of Nigeria, and African-Americans in Indianapolis found a high prevalence of Alzheimer’s disease amongst elderly Nigerians [24]. Similarly, Sokoya and Baiyewu found a higher incidence of geriatric depression among poor older Nigerians [25]. Although, studies that examine the impact of geriatric diseases on old-age poverty in Nigeria are rare, an important consideration from this body of evidence is that old-age diseases carry huge financial implications on household budgets in Nigeria [26]. For instance, one study of medical admissions of elderly patients at a teaching hospital in South Western Nigeria reported a higher demand for inpatient facilities, and a higher incidence of premature discharge due to the high financial costs among poor elderly Nigerians [27]. A second consideration from the literature is that research into the existence and drivers of CHE remain largely unexplored in Nigeria. These are compelling reasons to study the existence and determinants of CHE among urban elderly households in Nigeria, at least economically. From a policy perspective, understanding the key determinants of CHE would engender the financial protection of vulnerable elderly households in resource-scarce contexts. This paper seeks to investigate the key determinants of CHE in Urban Nigeria using probit regression analysis.

## Methodology

### The urban sample

The Nigerian General Household Survey (NGHPS) collected data on 5,000 households in two rounds in 2010. The NGHPS survey comprises of 1,620 urban households; of these households, a sample of 1,176 urban elderly households (defined as a household with an elderly person in the household who is ≥ 50 years old) is utilised for the study2. The NGHPS is a nationally representative survey of households with detailed information on consumption expenditure. Consumption Expenditure Surveys are more superior to the National Demographic Health Surveys due to its representativeness of different types of consumption expenditure data. Nigeria’s National Demographic Health Survey in 2008 [28] captures health information and expenditure for the adult working population only; 15–49 years old for women, and 15–59 years for men, limiting its usefulness for a study of elderly households. More importantly, consumption expenditure is arguably a better measure of living standards of households in developing countries, as demonstrated by empirical studies on developing countries [29, 30]. Deaton’s work on household surveys in developing countries documents extensively the reliability of consumption data over income. We refer readers to Deaton [29] for more additional information.

The NGHPS 2010 data is available on household heads by age, sex, household health spending, out-of-pocket payments, total consumption expenditure, food and non-food expenditure, household size, and region. Data on health was collected over the second round of the panel survey. It contains information on occupation, NHIS membership contributions, credit and savings, private health insurance, 12-month out-of-pocket health spending on all household members; while 12-month household expenditure was collected on a household basis. Consumption expenditure was collected over a one year period, and this was sourced from the first round of the panel survey. The difference between the first and second round was not significant so the issue of selection bias should not arise. Household size was adjusted for accordingly, and income per capita was then divided into four quintiles: 1- most poor, to 4 – richest. Education, self-reported health, and labour were collected on an individual basis; however, household income and health expenditure data were collected on a household level. OOP have been summed up based on individuals in each household and assigned to the household head, after adjusting for household size. The household health expenditure measure is based on all types of health spending during the survey period while OOP measure is based on hospitalisation and prescription costs.^2^

### Model specification and empirical strategy

The study applies a probit model to investigate the determinants of catastrophic health expenditures. The dependent variable is a binary outcome variable that is coded 1 or 0. The probability model is specified as:

1 $$ \Pr\ (CHE)={\beta}_o + {\beta}_1{x}_1+{\beta}_2{x}_2+\dots +{\beta}_k{x}_k + {\varepsilon}_i $$

Where Pr (*CHE*) is the probability of an observation (Y) being 1, where the dependent variable is coded 1 if the household incurs CHE and 0 otherwise, *β* is the coefficients, and *x*_1_ *to x*_*k*_ is a set of explanatory variables, *ε* is an error term that includes all other useful information.

Probit models estimate coefficients that provide useful information on the direction of the effect of the change. For instance, a negative coefficient suggests that the explanatory variable is less likely to be associated with our dependent variable, Pr (CHE), and vice versa, holding all other explanatory variables constant. Marginal effects measure the change of *x*_1_ *to x*_*k*_ on Pr (CHE).

We accept a 10 % of consumption expenditure CHE threshold, after testing the 5 %, 10 %, and 40 % thresholds. For brevity, we do not present results on the formal tests of alternative specifications in this paper.^3^ CHE was then estimated using the standard maximum likelihood (ML) techniques reported by O’Donnell et al. [30]. ML techniques produce asymptotically consistent standard errors, and converge to the most likely values that maximise the likelihood function [31]. The probit model allows us to model our binary dependent variable, and produce more consistent estimates compared to a linear probability model (LPM) which needs to be constrained to ensure that all values in the model lie within the (0,1) range [30]. More importantly, linear models can be problematic in interpreting interaction terms as explained by Ai and Norton, and Karaca-Mandic et al. [32, 33].

We also note the unresolved issue of sample size requirements of ML models in the literature. Econometricians have typically dismissed the sample size issue on the strength of the asymptotic qualities of ML models [34]. However, other applied researchers have made specific recommendations in their studies. Eliason has recommended that a sample size of more than 60 should be adequate [35]. Hart and Clark found that problems of inference begin to occur when the number of cases is less than 30, and in another study by the same authors, a sample size of 200, produced consistent estimates for the probit model [36]. Therefore, it is our belief that our sample size of 1,176 households is adequate for the requirements of the model.

To engender interpretations of the coefficients from the probit model, marginal effects of the regressors are also estimated. Standard errors have been estimated at the means using the conventional Delta method [37]. We re-estimate the marginal effect for ‘age_fem’ separately as a cross-partial derivative [32, 33]. In otherwords, the marginal effect of age of the elderly household head on CHE incidence, based on gender. Standard descriptive analyses are utilised to summarise our variables. All our analysis was done in STATA version 13.

### Robustness analysis

The conventional tests of robustness are estimated for consistency, and we arrive at the functional model in (2) below. After careful residual analysis, we found that all the variables significantly explained the model based on the z-scores. We introduce a continuous interaction term *(age_fem)* to capture age and gender effects, and found that it improved the model fit, and was positive and significant in the model (*p* < 0.01). The usefulness of interaction terms in non-linear models has been detailed in Ai and Norton [32] and Karaca- Mandic et al. [33]. The likelihood-ratio reports the joint significance of all of the coefficients, and has a p-value of 0.0000, indicating that our model is statistically significant with the inclusion of these predictors [31, 34]. The convention is to examine multi-collinearity in non-linear models based on the convergence criterion [38]. Our model converges at the 4^th^ iteration, providing support that multicollinearity is weak or absent.

### Variable selection

This section discusses the explanatory variables in our model and expected relationships. Prior studies on other developing countries offer some guidance as to the relative importance of the possible determinants of CHE.

One study by Somkotra and Lagrada, on Thai households found that poorer households, households with more elderly, incidence of chronic illness, and hospitalisation were positively related to CHE [12]. Brinda et al. reported that age of the household head, education, chronic illness, household size, and income were some of the drivers of CHE among Tanzanian households [10]. Similar findings have also been echoed in other CHE studies [1, 11, 39, 40].

Given the limited health insurance coverage in Nigeria, we would expect that for elderly households, having a hospitalised member, a member with an ADL (Activities of Daily Living) difficulty, more than one elderly, would all be positively related with the risk of incurring CHE. Health insurance has also been found to reduce financial risk of CHE among households in some African countries [4, 41]; therefore, we expect a negative relationship between enrolment in social health insurance and our outcome CHE variable.

With respect to household size, our expectation of a relationship could either be positive or negative. The hypothesis that, as household size increases, health expenditure levels increase, has been challenged by studies on household economics.^4^ These studies have shown that large welfare increases can actually result from economies of scale associated with increasing household size and resource pooling [42]. On the other hand, among Tanzanian households, larger households were reported to be one of the main drivers of CHE [10].

In addition, our data reveals that 51 % of urban elderly households are self-employed. We have noted earlier the difficulties in engaging in income-generation at older ages; therefore, it is reasonable to expect that elderly household heads in self-employment are less likely to incur CHE, as they probably have more resources at their disposal in comparison to those who are economically inactive. This expectation of a negative relationship with CHE is also linked to the education effects explained by Grossman’s well-established demand for health model—educated households are more likely to have healthier lifestyles and respond to health messages [43], therefore, less likely to incur catastrophic spending. Although, some CHE studies have confirmed this theory [10, 12, 44], others have found inconsistent evidence in other LMICs. Using the get Survey of Consumption Expenditures 2006 from Turkey, Yardim et al. found that education of the household head was not associated with CHE [40]. In relation to the influence of public health messages, one good example in Nigeria is the use of treated bednets to combat malaria, which may be good proxy indicator for health maintenance strategies in the household. Malaria is well-known for its financial burden on the household, and societal costs in Nigeria [45, 46], therefore, we would expect that those using treated bednets as a health promoting tool, would be less likely to incur CHE.

The study uses an ‘informal health care financing’ *ad hoc* measure to account for other aspects of health expenditure, besides out-of-pocket spending in our survey. The assumption that this measure is indicative of informal health care is characteristic of a developing country context where unorthodox health care is used alongside formal health care. However, for the purpose of the study, we assume that this aspect of health care is financed informally through friends and extended family members. Given the low levels of formal borrowing and prepayment scheme membership, this is a reasonable assumption. Using the Living Standard Measurement Surveys on 13 developing countries, Banerjee et al. found low levels of access to formal credit, and savings markets especially amongst the poor [47]. When all out-of pocket payment options have been exhausted, it is reasonable to assume that elderly households probably resort to informal financial support for health care costs. From a policy perspective, if this measure is negatively related to CHE, it may offer an opportunity to financially protect vulnerable elderly households through existing informal networks.

In addition, we noted previously, the prevalence of fee-paying private health services in Nigeria, and its financial implications on household budgets. We therefore, expect that households using private health facilities may have a higher health spending compared to those that use government health services in urban Nigeria. Very few studies analyse the role of private facilities use on CHE risk in developing countries, however, in 2009, Vaishnavi and Dash [48] found that 60 % of urban Indian households using private facilities were more likely to incur CHE compared to those that use public sector facilities.

In summary, we control for: household size, sex, and age of household head, proportion of elderly in the household. Our selected socio-economic factors are — education, occupation, income, informal healthcare financing measure, health insurance coverage, self-reported health status indicated by an ADL, specifically self-care difficulty, and hospitalisation.

Therefore, including all variables, our simple functional form takes the form:

2 $$ \Pr (CHE)={\beta}_{\mathrm{o}}+{\beta}_1age\_fem+{\beta}_2HHs+{\beta}_3Sex+{\beta}_4Age+{\beta}_5Ag{e}^2+{\beta}_6Eld1+{gamma}_1Edu+{gamma}_2Occu+{gamma}_3Phf+{gamma}_4Bednet+{gamma}_5Hosp+{gamma}_6Selfdiff+{gamma}_7NHIS+{gamma}_8Infs+{\varepsilon}_i $$

Where:

Our set of explanatory variables; *x*_1_ *to x*_*k*_ are: age_fem = age*fem; HHs = Household size; Sex = Sex (Female =1; Male = 0); Age = Age; Age^2^ = Age squared; Edu = Education of household head; Eldn_1_ = (has >1 elderly =1, 0 otherwise); Edu = (years of educ > 6 = 1, 0 otherwise); Occu = (self-employed = 1, 0 otherwise); Ph_f_ = (private health facility =1, 0 otherwise); Bednet = (B_1_ to B_4_, ref: B_1_:untreated net); Hosp = (has hospitalised member = 1, 0 otherwise); Selfdiff = has self-difficulty =1, 0 otherwise); NHIS = (without NHIS =1, 0 otherwise); Inf_s_ = (has informal support =1, 0 otherwise).

### Data analysis and results

Table 1 shows that 9.6 % of households face CHE. Using a statistical Chi-square test, we find significant differences across the income quintiles for the 10 % CHE threshold (*χ*^2^ = 14.819, *p* < 0.05). Table 2 presents descriptive statistics on all our variables of interest. Majority of the heads of households in the study sample were men. Mean age of household head is approximately 55 years with the highest being 102 years old. Less than half of household heads were women (female = 1). This finding is consistent with the 1991 Census data on elderly households in Nigeria [49]. The interaction term between age and female (*age_fem*) has a mean of 13.17 and ranges from 0 to 95. Table 2 Row 1 shows that average household size for urban elderly households is 4.88 (S.D = 3.12). The average size is not too surprising due to the urban context of the study, where families are typically known to be more nuclear [50]. Very few elderly live alone (*N* = 15) which conforms to studies on living arrangements on elderly people in Sub-Saharan Africa where co-residence with others is common [51, 52]. 68 % of urban elderly households do not use either an untreated or treated bednet to protect their households from malaria. Only 6 % of urban elderly households used treated bednets for more than 6 months. More than half of elderly household heads are educated to at least primary education. Less than 1 % of households are enrolled in the NHIS in our sample.

**Table 1:** Proportion of households with CHE at variable thresholds and by expenditure quintiles (*N* = 1176)

	Mean	Expenditure Quintile			
		Q1: Poorest	Q2	Q3	Q4: Richest
10 % of total household expenditure	9.61	14.63	10.54	6.46	6.50

Author’s analysis based on the NGHPS 2010 (urban elderly sample). Expenditure differences across the household quintiles are statistically significant at the for 10 % CHE threshold (*p* < 0.05)

**Table 2:** Summary statistics of variables of interest, Nigeria 2010

Notation	Variables of interest	Description	Mean	SD^a^	Min	Max
HHs	Household size	Household size	4.88	3.12	1	24
Sex	Sex:	Sex of the household head				
	Female	Female head dummy	0.22	0.42	0	1
	Male	Male head dummy	0.78	0.42	0	1
Age	Age	Age of household head	54.73	15.09	18.00	102.00
Age_fem	Age and Gender	Interaction term for age*gender	13.17	25.7	0	95
Edu	Education of household head	1: has attended school, 0 - otherwise	0.72	0.45	0	1
Eldn_1_	Proportion of households with >1 elderly member	1: has >1 elderly, 0 otherwise	0.52	0.50	0	1
Occu	Proportion of households that are self employed	1: head of household is self-employed, 0 otherwise	0.51	0.50	0	1
Ph_f_	Proportion of households using private health facilities	1: household uses private health facilities, 0 otherwise	0.08	0.27	0	1
Bednet	Health promoting measure – bednets type:	Proportion of households using bednets				
B_1_	Untreated bednet	1: yes, 0 otherwise	0.07	0.26	0	1
B_2_	Treated bednet use < 6 months	1: yes, 0 otherwise	0.19	0.39	0	1
B_3_	Treated bednet use > 6 months	1: yes, 0 otherwise	0.06	0.23	0	1
B_4_	No bednet	1: none, 0 otherwise	0.68	0.47	0	1
Hosp	At least one member has been hospitalised	1: yes, 0 otherwise	0.03	0.18	0	1
Selfdiff	At least one member has a self-care difficulty	1: yes, 0 otherwise	0.04	0.19	0	1
NHIS	Proportion of households without NHIS coverage	1: yes, 0 otherwise	0.60	0.49	0	1
Inf_s_	Proportion of households with informal financing support	1: yes, 0 otherwise	0.40	0.49	0	1

Author’s calculations based on the NGHPS 2010 data (*N* = 1176), urban elderly households sample

^a^S.D: standard deviation

**Table 3:** Probit model estimates of the determinants of CHE of urban elderly household in Nigeria

Dependent Variable = 1, has CHE at 10 % of consumption expenditure, 0 otherwise		
Log likelihood = −314.29794		
Determinants	Coefficient	Standard error
Household living standards: ref (1: poorest)		
2nd quintile	−0.222	(0.151)
3rd quintile	−0.549***	(0.162)
4th quintile	−0.754***	(0.187)
Household size	−0.074***	(0.018)
Female household head (ref: male)	−1.407**	(0.520)
Age_fem	0.023**	(0.008)
Age of household head	0.000	(0.021)
Education of household head (ref: no education)		
Household head is educated at least to primary education	0.349*	(0.143)
Proportion of households that are self employed	−0.238	(0.169)
Proportion of households with >1 elderly member	0.222	(0.116)
Proportion of households using private health facilities	0.027	(0.213)
Bednet use (ref: household uses untreated net)		
Treated bednet use < 6 months	0.450	(0.266)
Treated bednet use > 6 months	0.708*	(0.311)
No bednet	0.300	(0.250)
At least one member has been hospitalised	−0.131	(0.330)
At least one member has a self-care difficulty	−0.178	(0.290)
Proportion of households without NHIS coverage	0.108	(0.172)
Proportion of households with informal financing	−0.646***	(0.124)
Constant	−0.782	(0.699)
N	1140	
LR (chi2)	80.611	
Prob > chi2	0.0000	

Our model includes agesquared. We excluded region from the estimation as it is insignificant in the model. We introduce an interaction term “age_fem” to capture any effects that gender and increasing age has on CHE. Log likelihood converged on the 4^th^ iteration

NGHPS data, 2010 (urban elderly households sample)

Standard errors in parentheses. Significance levels: *p < 0.05 **p < 0.01 ***p < 0.001

There were 38 cases of hospitalisations, and 45 cases of self-care difficulties. These low numbers are hardly surprising, as elderly Nigerians have been known to be optimistic about their health status. In a study of elderly Nigerians, Baiyewu et al. reported that in a cohort of 951 elderly persons, 95 % of elderly people did not report any functional impairment [53]. We now turn to estimating our model. Table 3 presents the results of our estimated model.

### Coefficients of Probit models

This section presents the associative factors of CHE among urban elderly households in Nigeria. We interpret the probit model based on the direction of the effects of the coefficient estimates in Table 3. These estimates capture the values that maximise the log-likelihood function of CHE. We find support for the hypothesis that the risk of incurring CHE decreases with higher income (*p* < 0.001), and having access to informal health financing significantly reduces the risk of CHE (*p* < 0.05). Our probit model also reveals that larger household size was negatively related to CHE finding support for the economies of scale argument (*p* < 0.001). We found counterintuitive evidence that more educated households are more likely to incur CHE than less educated household heads (*p* < 0.05). We did not find support for the hypothesis that utilising treated bednets will reduce the risk of CHE.

Non-enrolment in health insurance (NHIS) was positively associated with the risk of CHE, whilst having a member who is hospitalised or with self-care difficulties was negatively related to the risk of CHE, however these were all not significant. Care must be taken in interpreting the results from these three independent variables due to the small number of cases—all less than 50. As Hart and Clark explained, 30 to 50 cases per independent variable would be required to avoid Type II problems caused by a small β to SE ratio, and to provide a larger test statistic which performs better [36].

### Marginal effects

Marginal effects measure the percentage changes in the probability of having a success in the dependent variable in response to a percentage change in the explanatory variable, all things being equal. The marginal effects are approximations based on an additive scale, and are useful in interpreting the partial effects of the coefficients of the probit model.

Table 4 presents the average marginal effects of our predictors in the probit model. The linear extrapolation for the dummy variables is slightly different for the continuous variables in our model like age and household size. This is because a change in a binary variable (0 to 1 or vice versa) is indicative of a 100 % change in probability, and this is all that can be inferred. For the continuous variables, we can extrapolate based on a specified percentage. For instance, a 10 % increase in household size will result to a 0.1 % increase in the probability of incurring CHE.

**Table 4:** Average marginal effects of the significant probit model estimates

Dependent Variable = 1, has CHE at 10 % of consumption expenditure, 0 otherwise		
Determinants	dy/dx	Standard error
Household living standards: ref (1: poorest)		
2.quintile_c	−0.0444	(0.0306)
3.quintile_c	−0.0935***	(0.0283)
4.quintile_c	−0.115***	(0.0291)
Household size	−0.0110***	(0.00269)
Female household head (ref: male)	−0.210**	(0.0783)
Education of household head (ref: no education)	0.0522*	(0.0213)
Bednet use (ref: household uses untreated net)		
Treated bednet use < 6 months	0.0594	(0.0305)
Treated bednet use > 6 months	0.110*	(0.0497)
No bednet	0.0358	(0.0253)
Proportion of households with informal financing	−0.0966***	(0.0188)

Standard errors in parentheses

NGHPS data, 2010 (urban elderly households sample)

*p < 0.05 **p < 0.01 ***p < 0.001

With respect to our income groups, a 100 % increase in the number of those that are in the richer quintiles (3rd and 4th) will probably lead to an increase in incurring CHE by 9.4 % to 11.5 % respectively. Similarly, increasing the number of educated heads by 100 % will increase the probability of incurring CHE by 5.2 %. In comparison to men, increasing the number of women by 100 % in relation to men would reduce the probability of incurring CHE by 21 %. However, a 100 % increase in elderly females will result in 0.4 % increase in the probability of incurring CHE. Table 5 presents marginal effects for our interaction term, age_fem. Age and gender effects are however minimal, a 10 % marginal increase in age will result in a less than 1 percentage point increase in the probability of incurring CHE for female heads, than male heads..

**Table 5:** Average marginal effects of “age_fem” probit model estimate

age	dy/dx^a^	Standard error
1. Male	-.000331	(.004110)
2. Female	-.000083	(.001029)

NGHPS data 2010 (urban elderly households sample)

^a^dy/dx is the discrete change of the gender dummy variable from 0 to 1. Difference (2) – (1) = 0.00025, the average marginal effect of age on CHE with respect to gender. Standard errors in parentheses: *p < 0.05 **p < 0.01 ***p < 0.001

We also find that a 100 % increase in the number of those with informal support for healthcare costs reduced the probability of incurring CHE by 9.6 %. Increasing the number of educated household heads and those using treated bednets by a 100 % change in probability increased the probability of incurring CHE by 5 % and 11 % respectively. These findings could be interpreted that those that are educated are likely to spend more on their health increasing the risk of financial catastrophe, a finding that is consistent with some studies in the literature [4, 12].

## Discussion

The results presented throughout this paper show the effects of socio-economic determinants on the probability of CHE among elderly households. We discuss each finding in turn, and attempt to reconcile the findings. We also identify areas of further research.

### Poor elderly households versus richer elderly households

From our findings, richer households are less likely to incur CHE compared to poorer households (*p* < 0.001), which conforms to the literature on CHE in Africa. Given the regressive nature of fees in Nigeria’s health system, where both rich and poor households pay the same amount for health care as well as the limited coverage of social health insurance amongst elderly households, income represents a key driver of CHE amongst households in urban Nigeria.

### In search of gender effects

Social roles play a key role in determining gender equity of health in many developing countries [54]. Economic theory suggests that a person’s propensity to seek health care is dependent on the costs and the utility perceived to be derived from such health care [43, 55]. Average marginal effects of the gender (female = 1) were negative and significant; with respect to the *age_fem* interaction term on the probability of incurring CHE, average interaction effects of an increase in age differed between men and women, with women more likely to incur CHE with age, although these effects were small and insignificant. However, these findings also need to be interpreted in the context of Africa. There is evidence that African women, in particular spend less on health due to low financial status compared to their male counterparts. For instance, Russell reported that women in developing countries continue to work during periods of illness as they are unable to afford the opportunity costs of illness [56]. Wouterse also found gender bias in health care spending in favour of men in Burkina Faso [57]. We suggest a gendered panel study on the determinants of health expenditure in Nigeria in revealing health spending patterns of elderly women in Nigeria, which will further explain the inconsistent gender effects on CHE in this study. The low levels of OOP for female-headed elderly households in our study compared to male heads in Table 6 support the notion that there are gender differences in health spending in Urban Nigeria. However, the question regarding the reasons for such differences in direct-out-of-pocket payments remain unanswered.

**Table 6:** Mean and Standard deviation of OOP and health expenditure by gender, in Nigerian Naira and US Dollars

Mean OOPs per capita	Mean	SD	Min	Max	$
Mean out of pocket payments per capita (total sample)	2333.88	5460.77	13.00	72500.00	15.56
Mean out of pocket payments per capita (female)	2011.01	4263.55	16.25	31600.00	13.41
Mean out of pocket payments per capita (male)	2424.61	5753.10	13.00	72500.00	16.16

Author’s calculations based on the NGHPS data (*N* = 1176). Urban elderly households sample. All figures are in Col 1–4 are in Nigerian Naira. Col 5 in US dollars. (Conversion: $1 = 150 Nigerian Naira, 2010)

### Elderly size effect

The economic dependence of elderly people in Nigeria, following a geriatric illness has been well documented in the literature [23, 25, 53, 58–61]. Returning to Tables 3 and 4 we find a positive but insignificant elderly size effect. Elderly size effects are perhaps not as prominent in the Nigerian case compared to studies in Asian countries [11, 12]. Perhaps, because elderly Nigerians contribute to their households economically, often working beyond retirement age [18]. Our univariate analysis in Table 7 revealed that households with more working age members were less likely to incur CHE (*p* < 0.01), all things being equal, suggesting that both household composition and household size are important determinants of CHE in Urban Nigeria.

**Table 7:** Univariate analysis of CHE and having more than one working age member

Dependent variable: Pr (CHE)	Coefficient	Robust SE
Working age members > 1	−0.074*	0.030

NGHPS data, 2010 (urban elderly households sample). Standard errors in parentheses

Significance levels: *p < 0.05 **p < 0.01 ***p < 0.001

### Effects of education

Education is a key driver of CHE among urban elderly households in Nigeria. However, contrary to Grossman’s theory [43], more educated households are associated with a 5.2 % increase in the likelihood of incurring CHE. The theory typically suggests that more educated households are likely to be more efficient in maintaining health over time, and hence are less likely to be vulnerable to serious health conditions which lead to catastrophic health spending. However, one possible explanation for our finding is that urban elderly households in Nigeria may be inefficient in their health spending and use of modern medicine. However, without data on the pricing of medical services and longitudinal data to confirm this possibility, our results are to be interpreted cautiously.

### Informal safety nets and CHE

Healthcare financing from informal sources reduced the likelihood of incurring CHE (*p* < 0.001). One interpretation is that those with access to informal networks probably have a higher capacity of coping with significantly high health payments, thereby delaying the catastrophic effects on the households. We are limited by our data to identify the nature of such informal support. We expect that the implications would differ somewhat depending on whether informal health financing is in the form of a loan or a gift, and whether the effect is consistent over time. Nevertheless, our findings of a negative relationship are consistent with the literature [48, 62].

### Use of treated bednets

In our study, the use of treated net increased the likelihood of incurring CHE in comparison to those using untreated bednets (*p* < 0.05). The provision of free treated bednets in mitigating the high incidence of malaria, and its societal costs was first proposed by the WHO to African countries as a poverty alleviation measure [46, 63, 64]. Since 2009, treated bednets are available to all Nigerians at no cost [65]. Therefore, it is surprising that compared to those households using untreated bednets; treated bednets increased the risk of CHE. An impact evaluation study on the effectiveness of the policy of free treated bednets is probably needed to provide a more robust explanation of the counterintuitive evidence found in the study. We suggest that further research be undertaken in this regard.

### Study limitations

In addition to some of the issues identified above, our study utilises a cross-sectional design. Therefore, causal associations about the likelihood of CHE and its determinants cannot be inferred. As this study is based on elderly households in urban Nigeria, generalisations to rural households in Nigeria or in other African settings would be inaccurate. Data on geriatric illnesses may yield stronger results compared to self-reported measures of hospitalisation and ADL difficulty. More importantly, we recognise that there are other possible drivers of CHE that are not considered in this study, for instance, proximity to health services. If elderly households have to travel far distances, it may increase or decrease health spending, all things being equal. Lastly, while our ‘informal health financing’ measure is a subjective term that fits the cultural context in Nigeria, it assumes stronger communal relations, than is probably the norm in an urban West African setting.

### Implications for policy

The identified determinants of CHE which place urban elderly households at risk can be addressed through policies that help support household budgets. In Nigeria, attaining healthcare equity remains a primary objective of Nigeria’s health policy [66]. In 2006, Nigeria’s health policy reform extended the National Health Insurance Scheme (NHIS) to protect households from CHE and to ensure universal health coverage. Currently, only 3 % of the population are currently enrolled in the scheme [17]. However, it is our belief that because Nigeria’s NHIS is in its early stages, it is easy to modify its current provision to include disadvantaged populations. Administratively, Nigeria’s current NHIS allows for such expansion and integration to deliver benefits for elderly households in Nigeria.

We propose two approaches to achieving better coverage for elderly people: First, the NHIS’s Vulnerable Groups Program could be modified to include elderly people from the age of 50 years old. Secondly, given that a considerable number of urban elderly are engaged in self-employed work, in the short term, outreach programmes can be instituted to encourage enrolment into the recently implemented self-employed NHIS [17, 41]. Education levels are clearly influential for engaging people to understand the importance of health insurance and to understand the application procedures involved [41, 67]. Therefore, community-based representatives can be appointed to help elderly people navigate the enrolment processes in prepayment programmes. Studies on other African countries have shown that targeting vulnerable groups using pre-payment schemes works in reducing the incidence of CHE [4, 41, 62]. One good example is Ghana, which has now achieved 54 % comprehensive health coverage of its population, and only 2 esources are shared by the family to meet the needs of elderly members [68, 69]. With age, the dependence on family increases for poor and economically inactive elderly people. This overdependence on extended family and friends for health care costs in impoverished urban areas, can increase the economic vulnerability of elderly people and their households [69, 70]. In a study of elderly Nigerians, Akanji et al. [26] found that many elderly people have to depend on family to bear the burden of medical care when financial resources are low. Therefore, we support the proposal by some health advocates in Nigeria for the establishment of a health fund to subsidise health care for elderly people. The health fund financed through tax revenue on luxury goods would support policy efforts to provide healthcare insurance coverage to vulnerable groups including poor elderly citizens in Nigeria [71]. Funding membership premiums for elderly people in this way will encourage enrolment and reduce out-of-pocket spending. This approach also avoids the well-documented complications of anti-poverty cash transfers paid to elderly households [72, 73].

## Conclusions

This paper has investigated the existence and determinants of CHE among urban elderly households in Nigeria. Clearly, more attention is needed to reduce CHE amongst poor urban elderly households. Extending the national health insurance scheme to provide coverage for elderly people would reduce the financial burden on households. The government should fund membership premiums for elderly people through the proposed special health fund, to encourage enrolment and reduce the risk of catastrophic spending. The policy recommendations in this paper may also be relevant in other urban African contexts.

## Abbreviations

CHE: Catastrophic Health Expenditure
OOP: Out of pocket health payments
LMICs: Low and Middle Income Countries
WHO: World Health Organisation
NHIS: National Health Insurance Scheme, Nigeria
ITN: Insecticide treated bednets or treated bednets
ML: Maximum Likelihood
ADL: Activities of Daily Living

## Footnotes

1 By our study’s age-cut off definition of 50 years and above, we expect the elderly population estimate to be higher.

2 We take the position that ageing is likely to begin from 50 years old for urban Nigerians to prevent losing valuable insights on elderly groups in Nigeria. The convention is to adopt the criterion in many western literature of using 60 or 65 years to define who the elderly are. Apart from a few recent epidemiological studies and the WHO reports on Ageing in Africa – most studies follow convention. However, there is contrary evidence to support a lower ageing cut-off point for countries with low life expectancies. Key stakeholders in the WHO’s minimum data set project (1995–2003) presented strong arguments for the development of a separate criterion for Africa and suggested 50 or 55 years as the cut-off point for the elderly [74]. This communiqué continues to influence the WHO’s research studies on ageing in Africa (for instance, The World Health Organization (WHO)'s Study on Global Ageing and Adult Health (SAGE) – a longitudinal study of ageing and older adults). Moreover, in comparison with other LMICs, Nigeria’s life expectancy is still relatively low at 48 years old. This provides a strong basis for applying an age -adjustment for any study of elderly people in Nigeria. Our preliminary data analysis revealed similar physical functioning characteristics of those aged 50–59 and 60 and above in the NGHPS 2010 data adding more support for the definition of contextual definition of ageing in Africa in line with the WHO’s findings.

3 Results on the alternative specifications of CHE thresholds of 40 % of non-food expenditure can be furnished upon request.

4 Past research on the relationship between household size and CHE report mixed results. On one hand, some studies argue that larger households will incur larger health out-of-pocket payments because there is higher health demand amongst this group. This demand-side effect may push health spending higher to resulting to CHE. Therefore, in this case these larger households are more likely to report high levels of health spending leading to CHE. There is also another possible explanation: larger households may have fewer resources to spend on health in the first place because they are too poor to afford health care, and in this case are more likely to divert resource to subsistence rather than seek medical care therefore incurring low levels of health spending. We tested both scenarios. We do not present full results here but larger households (those with household size of greater than the average of 8) incurred low levels of health spending making them less likely to incur CHE in comparison to smaller households. This difference between the mean OOP for bigger and smaller households is significant (*p* = 0.0086). However, poorer elderly households tend to have higher household sizes in comparison to richer elderly households, and we found this difference to be statistically significant (*p* =0.0001).
