# Erratum to: Investigating determinants of catastrophic health spending among poorly insured elderly households in urban Nigeria

**DOI:** 10.1186/s12939-015-0241-4

**Published:** 2015-10-26

**Authors:** Olumide Adisa

**Affiliations:** School of Sociology, and Social Policy, University of Nottingham, University Park Campus, Nottingham, NG7 2RD UK

The original version of this article [1] unfortunately contained a mistake. In the subsection titled “Implications for policy” part of the text was incorrect. It read “One good example is Ghana, which has now achieved 54 % comprehensive health coverage of its population, and only 2 esources are shared by the family to meet the needs of elderly members [68, 69].” The corrected text can be found below:

“One good example is Ghana, which has now achieved 54 % comprehensive health coverage of its population, and only 27 % of health spending is financed out-of-pocket [41]. Strengthen safety nets: In the Nigerian context, household resources are shared by the family to meet the needs of elderly members [67, 68].”

The original article was corrected to reflect this.
